# Associations between changes in physical activity and perceived social exclusion and loneliness within middle-aged adults – longitudinal evidence from the German ageing survey

**DOI:** 10.1186/s12889-023-15217-6

**Published:** 2023-02-07

**Authors:** Linda Baumbach, Hans-Helmut König, André Hajek

**Affiliations:** Department of Health Economics and Health Services Research, University Medical Center Hamburg-Eppendorf, Martinistr. 52, 20246 Hamburg, Germany

**Keywords:** Physical activity, Loneliness, Social exclusion, Social isolation

## Abstract

**Background:**

Previous research showed negative associations between physical activity and loneliness in older adults. However, information on associations among middle-aged adults is scarce. In this prognostic factor study, we investigated if starting or stopping to follow the WHO physical activity recommendations was associated with changes in perceived social exclusion and loneliness in this age bracket.

**Methods:**

We used longitudinal representative data of participants aged 40 to 64 years from the German Ageing Survey waves in 2014 and 2017 (analytical sample = 4,264 observations, 54% women). Perceived social exclusion was investigated with the scale from Bude and Lantermann. Loneliness was quantified with the 6-items loneliness scale from De Jong Gierveld. Information from the International Physical Activity Survey items on the time spend in moderate and vigorous physical activity per week was dichotomized. Participants were coded as either following or not following the WHO´s physical activity recommendations of spending at least 150 min of moderate, 75 min of vigorous or an appropriated combination of physical activity per week. We investigated the within (individual) association between starting and stopping to follow WHO´s physical activity recommendations and perceived social exclusion as well as loneliness in asymmetric fixed effects regressions. Analyses were adjusted for age, marital status, employment status, social-network size, general self-efficacy, depressive symptoms, self-rated health, BMI, comorbidities, and physical functioning (SF-36).

**Results:**

Stopping to follow the physical activity recommendations from the WHO was associated with perceived social exclusion (ß= 0.09 p = 0.04) but not with loneliness (ß=-0.01, p = 0.71). Starting to follow the WHO physical activity recommendations was neither associated with social exclusion (ß=-0.02, p = 0.54) nor with loneliness (ß=-0.01, p = 0.74) in adjusted asymmetric fixed effects regressions.

**Conclusion:**

In middle-aged adults, longitudinal associations were found for physical activity and perceived social exclusion. Perceived social exclusion may be prevented by maintaining at least 150 min of moderate physical activities per week, which is the WHO physical activity recommendation. Future research should investigate moderators and mediators in the association between physical activity and social exclusion as well as loneliness.

## Key points:

- First study concentrating on the within individual association between physical activity and perceived social exclusion and loneliness among middle-aged adults.
- First study, focusing on the impact of the physical activities change direction in the associations with perceived social exclusion and loneliness.
- Adults who stopped to follow the WHO physical activity guidelines experienced an increase in perceived social exclusion.

## Introduction

Loneliness is “the unpleasant experience that occurs when a person’s network of social relations is deficient in some important way, either quantitatively or qualitatively” [1]. Thus, people with few social contacts do not necessarily feel lonely and vice versa. Feeling lonely is related to the entirety of an individual´s existing interpersonal relationships and not context or situation specific. In contrast, perceived social exclusion refers to the feeling of being excluded from the society which is a specific context. The subjective experience of social exclusion raises from feeling not being able, allowed, or welcomed to participate in society [2].

Perceived social exclusion and loneliness are associated with physical, mental, and social health components [3–7]. Increased perceived social exclusion is observed in individuals with lower income and reduced actives due to financial reasons [2]. In particular, depressed individuals suffer from loneliness and it is a risk factors for mortality and reduced quality of life [3, 8]. To address adverse health outcomes of perceived social exclusion and loneliness, studies investigated relevant determinants [6, 7, 9–11]. A review highlighted that being female and not being married are both associated with loneliness. Further associations with higher loneliness were found for poor income, lower educational levels, living alone, low quality relationships, poor self-rated health, and poor functional status [11].

Physical activity could be an additional determinant of perceived social exclusion and loneliness. It is defined as any bodily movement, independent of intensity or intention [10, 12]. It is recommended as prevention and treatment for several diseases, which underlines its importance for a healthy living at all stages of life [13, 14]. The WHO (Word Health Organization) recommends: “Adults should do at least 150–300 minutes of moderate-intensity aerobic physical activity; or at.

least 75–150 minutes of vigorous intensity aerobic physical activity; or an equivalent combination of moderate- and vigorous-intensity activity throughout the week, for substantial health benefits.”

Associations between physical activity and perceived social exclusion and loneliness are of interest as initiating physical activity interventions could prevent long-term loneliness and consequent adverse health outcomes such as depression. An association with loneliness is expected since performing physical activities leads to higher self-efficacy [15, 16], which in turn is also associated with reduced loneliness [11]. Differentiating between loneliness and perceived social exclusion is of importance since to ideally address these two constructs with physical activity different interventions might be indicated.

Thus far, there is a lack of studies investigating the association between physical activity and perceived social exclusion. On the other side the association between physical activity and loneliness has been investigated in several studies – as summarized by a systematic review [10]. Most studies used a cross-sectional study design, which is restricted in causality and in providing the within individual association. A review found that the majority of these cross-sectional studies treat physical activity as independent and loneliness as dependent variable, however some studies also assumed the reversed causality [10]. Underling theoretical models for the association are scare, however intervention studies indicate a positive effect of physical activity interventions on mental health which likely include loneliness in adolescence and children [17], but no effect in older adults on loneliness [18]. Thus, the timing of a physical activity intervention might influence the effect on loneliness. However, a related systematic review identified, all included studies to focused on adolescence or older individuals, and all longitudinal studies dealt with individuals in adolescence only [10]. Thus, so far, the association for middle-aged adults (40 to 64 years) was neglected in longitudinal studies, despite its potential of indicating an appropriate window for interventions. Loneliness and perceived social exclusion are increasing with age and long-term loneliness may lead to depression. If an association between loneliness, perceived social exclusion and physical activity is already present in middle-aged adults, interventions to maintain or increase physical activity as prevention of long-term perceived social exclusion and loneliness later in life, should probably be initiated in this age period. This would assist successful ageing and reduce adverse health outcomes.

To close this research gap, we aimed to investigate longitudinal (within individual) associations between physical activity and perceived social exclusion and loneliness in middle-aged adults.

Based on the results for adolescence and older adults, we presume a negative relation between physical activity and perceived social exclusion and loneliness [10, 19, 20].

## Methods

### Data source

For this prognostic factor study, we used data from the cross-sectional and longitudinal German Ageing Survey (DEAS – “Deutscher Alterssurvey”). The survey aims at monitoring the aging process of community-dwelling adults in Germany. The survey is funded by the German Ministry for Family affairs, Senior citizens, Women and Youth (BMFSFJ) and carried out by the German Centre of Gerontology (DZA – “Deutsches Zentrum für Altersfragen”.

Since 1996 every three to six years, German citizens above the age of 40 (“second half of life”) sampled from German registries within municipalities are invited to participate or reparticipated at an interview and to fill out a questionnaire. The interview covers information on socio-economic circumstances, living conditions, and other topics related to aging. It is conducted by trained interviewers using a standardized interview guide. The questionnaire extended the data from the interview with information on subjective attitudes, psychological topics, and health information. The specific topics in the interview and the questionnaire are constantly updated, to respect emerging evidence and to better cover the desired information.

In the present study, data from 2014 (wave 5) and 2017 (wave 6) were included as the independent variable of interest was first added in 2014 into the data collection. The waves include information from 10,324 to 6,626 German citizens in 2014 and 2017, respectively. The response-rate of 27% for first-time participants in 2014 is comparable to other German survey studies but lower in comparison to European survey studies [21, 22]. Denial of re-participation was most frequently, if at all, justified with limited time and health restrictions [23]. Matching the aim of the study, we only included participants between 40 and 64 years, to evaluate if this age bracked is a target for physical activity interventions.

### Variables

#### Dependent variable

Perceived social exclusion and loneliness, our dependent variables of interest, were measured with standardized assessments in the DEAS.

To evaluate perceived social exclusion the 4-item-scale from Bude and Lantermann (2006) was used [24]. Participants rated each of the following items on a 4-point-scale from strongly agree [1] to strongly disagree [4]: “I feel excluded from society”, “I am worried to be left behind”, “I feel that I am left out” and “I feel like I do not really belong to society”. Based on the responses of at least two items a mean score was built with higher values indicating higher perceived social exclusion. Thus, it was treated as a continuous variable, which is in line with previous research [25]. The Cronbach’s alpha in the current study was 0.89.

To evaluate loneliness, the reduced 6-items scale De Jong Gierveld loneliness scale (2006) was used [26]. Participants responded to each of the items on a 4-point-scale from strongly agree [1] to strongly disagree [4]. Based on at least three responses to the six-items a mean score between one and four was calculated, with higher values indicating higher loneliness. The current studies Cronbach’s alpha was 0.85.

#### Independent variables

The independent variable of interest was physical activity, in a dichotomous why of following or not following physical activity recommendations of the WHO. Questions relating to those from the “International Physical Activity Survey” (IPAQ) were utilized [27, 28]. Three questions, each addressing one intensity of low, moderate, and vigorous physical activity, with six response options were asked. Each question was underpinned with examples of the respected physical activity intensity and related changes in breathing. The six response options were: “daily”, “several times per week”, “once a week”, “1–3 times per month”, “less frequent”, or “never”. Those participants, who answered at least “once a week” were additionally asked for the hours and minutes spent in the respected physical activity intensity. If participants answered less than once a week, the respected time spend in this physical activity intensity was coded with zero. In line with the IPAQ, we excluded participants who indicated, that they spend more than 16 h in sum of all physical activities per mean day (n = 49 in 2014, n = 25 in 2017) [29]. This exclusion is based on the IPAQ assumption that people spent on average 8 h sleeping, thus they cannot be physically active for more than 16 h per day. In line with the WHO recommendations, we dichotomized physical activity into following or not following the WHO physical activity recommendations of at least 150 min of moderate, 75 min of vigorous, or an appropriate combination of these two physical activity activities per week [30]. In consequence, since vigorous activity counts twice, the time spend in these activities was doubled and added to the time spend in moderate physical activities per week – low physical activities are not respected. Thus, an individual who stated to perform 30 min of vigorous and 90 min of moderate physical activities per week, spent a sum of 150 min (2 × 30 min + 90 min) in physical activities and was coded as following the physical activity recommendations.

#### Covariates

We adjusted our models for ten potential confounding variables, since omitting them could lead to spurious effects. Matching our asymmetric fixed effects regression analyses, all these variables were time-varying. We included socioeconomic, psychological, and health-related time-varying factors as covariates. Socioeconomic factors considered were age, marital, and employment status, as well as the social-network size. The psychological factors included were general self-efficacy and depressive symptoms. Finally, self-rated health, body mass index (BMI), sum of morbidities, and physical functioning were entered as health-related factors.

The marital, as well as the employment status were investigated with a multiple-choice question. Regarding the martial status, it was distinguished between “being married and living together”, “being married and living apart”, “divorced”, “widowed”, and “single”. Employment status was categorized into being “employed”, “retired”, and “other: not employed”. The social network size was determinant with the question: “We now want to look at people who are important to you and who you maintain regular contact with. These can include co-workers, neighbours, friends, acquaintances, relatives, and members of your household. Which people are important to you? If there are several, please just name the eight most important. Please give me these people’s first names and the first letters of their last names.” If participants had more than eight persons to mention, to avoid questionnaire load, they were only asked for the additional number of persons. However, any network size > 8 was coded as 9.

General self-efficacy was obtained with responses to five out of the eight statements of the scale from Schwarzer and Jerusalem [31, 32]. The reduction in questions was performed for the DEAS in corporation with Ralf Schwarzer. Each statement was rated by the participants from 1 = not at all true to 4 = exactly true. Based on the answers to the five statements a score was built by averaging the results from the individual questions. Low values indicate low and high values high self-efficacy. Depressive symptoms were indicated from 0 = low to 45 = high, by utilising the 15-item version of the Center for Epidemiological Studies Depression Scale (CES-D).

The self-rated health was obtained with a single-item question with response options from 1 = very good to 5 = very bad. The BMI was calculated based on self-reported height and weight. The range for the sum of morbidities goes from 0 to 11. Participants indicated to either have or do not have the single following conditions: (1) Cardiac and circulatory disorders, (2) Bad circulation, (3) Joint, bone, spinal or back problems, (4) Respiratory problems, asthma, shortness of breath, (5) Stomach and intestinal problems, (6) Cancer, (7) Diabetes, (8) Gall bladder, liver or kidney problems, (9) Bladder problems, (10) Eye problems, vision impairment, and 11) Ear problems, hearing problems. Physical functioning was obtained with the short SF-36 health survey (subscale physical functioning) [33]. The related score ranges from 0 = low to 100 = high physical functioning.

### Statistical methods

Descriptive statistics are provided as mean and standard deviation (SD) or number and percentage as appropriate. Afterwards we investigated the correlations of our variables. To investigate longitudinal association between physical activity and perceived social exclusion as well as between physical activity and loneliness on an individual level, we used asymmetric fixed effects regression models [34]. In these models, associations within an individual are evaluated on complete cases. Thus, the influence of unobserved variables is reduced, as the estimates of the fixed effects models are not biased by time constant variables (both, observed and unobserved). However, these constant variables, like sex cannot be included in the models (as main effects). The asymmetric term in the fixed effects regression models highlight, that differences in the effect estimate depending on the direction of change of the independent variable are investigated [34]. Thus we investigated if a participant (a) started or (b) stopped to follow the physical activity recommendations from the WHO from the year 2014 to 2017. The models were performed with and without adjustments for the covariates. The level of statistically significance was set at p < 0.05. The analyses were performed in STATA 16.1 (StataCorp., College Station, Texas, USA).

## Results

Our analytical sample based on the adjusted analyses of perceived social exclusion includes 4,264 observations. The samples mean age was 55 years, 54% were females and most of the participants were married and lived together with their partner. Further characteristics of the participants are given in Table 1.

**Table 1:** Characteristics of the 4,264 included observations in the adjusted analyses on perceived exclusion

Age, mean (SD), range 40–64 years	54.9 (5.8)
**Females, n (%)**	2,303 (54.0)
**Marital status, n (%)**	
Married, living together	3,121 (73.2)
Married, living apart	76 (1.8)
Divorced	468 (11.0)
Widowed	165 (3.9)
Single	434 (10.2)
**Employment status, n (%)**	
Employed	3,312 (77.7)
(early) Retired	423 (9.9)
Other (not employed)	529 (12.4)
**Social-network size, mean (SD)**, from 0 to 9	5.5 (2.7)
**Self-efficacy, mean (SD)**, from 1 to 4 with higher values indicating higher self-efficacy	3.1 (0.4)
**Depressive symptoms (CES-D), mean (SD)** from 0 = no depressive symptoms to 45 = severe depressive symptoms	6.8 (6.3)
**Self-rated health, mean (SD)**, from 1 = very good to 5 = very bad	2.4 (0.8)
**Body Mass Index (kg/m²), mean (SD)**	27.0 (5.0)
**Sum of morbidities, mean (SD)**, from 0 to 11	2.0 (1.7)
**Physical functioning subscale of the SF-36, mean (SD)**, from 0 = worst to 100 = best	87.4 (18.7)
**Followed the WHO physical activity recommendations, n (%)**	3,503 (82.2)

CES-D = The Center for Epidemiological Studies Depression Scale, MET = metabolic equivalent task, SD = Standard deviation, SF-36 = 36-Item Short Form Survey

In Table 2 the correlations of our variables are presented. Our dependent variables had a moderate correlation coefficient of 0.56.

**Table 2:** Correlations of our variables

	Perceived social exclusion	Loneliness	Age	Social-Network size	Self-efficacy	Depres-sive Symptoms	Self-rated health	Body-Mass-Index	Sum of Morbi-dities	Physical function-ing
Perceived social exclusion	1.00*									
Loneliness	0.56*	1.00*								
Age	0.01	-0.03	1.00*							
Social-Network size	-0.11*	-0.15*	-0.03	1.00*						
Self-efficacy	-0.45*	-0.44*	-0.01	0.06*	1.00*					
Depressive Symptoms	0.39*	0.35*	-0.00	-0.07*	-0.36*	1.00*				
Self-rated health	0.28*	0.23*	0.11*	-0.09*	-0.26*	0.46*	1.00*			
Body-Mass-Index	0.10*	0.08*	0.07*	-0.03	-0.03	0.11*	0.24*	1.00*		
Sum of Morbidities	0.26*	0.22*	0.18*	-0.04	-0.20*	0.33*	0.45*	0.26*	1.00*	
Physical functioning	-0.25*	-0.17*	-0.15*	0.07*	0.24*	-0.40*	-0.57*	-0.28*	-0.44*	1.00*

* Bonferroni-adjusted significance levels of ≤ 0.05.

All cases included in the asymmetric fixed effects regression models needed to experience a change in the independent variables. It is worth noting that 247 individuals stopped, and 254 individuals started to follow the WHO physical activity guidelines (from the year 2014 to the year 2017).

The estimates from the adjusted models are presented in Table 3.

In the unadjusted analyses stopping (ß= 0.09, p = 0.03) but not starting (ß= -0.03, p = 0.41) to follow the WHO physical activity recommendations was associated with a change in perceived exclusion. The adjusted analyses confirmed these findings for stopping (ß=0.09, p = 0.02) and starting (ß=-0.02, p = 0.54) to follow the physical activity recommendations of the WHO.

In unadjusted analyses on loneliness, neither starting (ß= -0.01, p = 0.74) nor stopping (ß= -0.01, p = 0.71) to follow the WHO recommendations was significantly associated with changes in loneliness, this observation was as well confirmed in the adjusted analyses (Table 3).

**Table 3:** Estimates of the adjusted asymmetric fixed-effects regressions

	Coefficients of perceived social exclusion	Coefficients of loneliness
**Start following WHO physical activity recommendations**	-0.02 (0.04) **[-0.10; 0.05]**	-0.02 (0.03) **[-0.08; 0.05]**
**Stop following WHO physical activity recommendations**	0.09* (0.04) **[0.01; 0.12]**	0.02 (0.03) **[-0.05; 0.08]**
**Age**	-0.00 (0.00) **[-0.01; 0.01]**	-0.01 (0.00) **[-0.12- 0.00]**
**Marital status** (Ref.= married, living together)		
Married, living apart	0.36+ (0.19) **[-0.02; 0.74]**	0.16 (0.14) **[-0.11;0.42]**
Divorced	0.06 (0.10) **[0.13; 0.25]**	0.07 (0.07) **[-0.08; 0.21]**
Widowed	-0.17 (0.13) **[-0.42; 0.07]**	-0.06 (0.09) **[-0.23;0.12]**
Single	-0.05 (0.09) **[-0.23; 0.14]**	-0.06 (0.08) **[-0,21; 0.10]**
**Employment status**, (Ref.= Employed)		
(early) Retired	-0.04 (0.05) **[-0.14; 0.05]**	-0.01 (0.04) **[-0.08; 0.06]**
Other	0.10+ (0.05) **[-0.00; 0.19]**	0.05 (0.03) **[]-0.02; 0.12**
**Social-network size**, (from 0 to 9)	-0.00 (0.00) **[-0.01; 0.00]**	-0.00 (0.00) **[-0.01; 0.00]**
**General self-efficacy** (from 1 = low to 4 = high)	-0.22*** (0.04) **[-0.29; -0.15]**	-0.23*** (0.03) **[-0.30; 0.17]**
**Depressive symptoms (CES-D)** (from 0 = no depressive symptoms to 45 = severe depressive symptoms)	0.01** (0.00) **[0.00; 0.01]**	0.00* (0.00) **[0.00; 0.01]**
**Self-rated health** (from 1 = very good to 5 = very bad)	0.04* (0.02) **[0.00; 0.07]**	0.03* (0.02) **[0.00; 0.06]**
**Body Mass Index (kg/m²)**	0.00 (0.01) **[-0,01;0.02]**	-0.01 (0.01) **[-0.02;0.00]**
**Sum of morbidities** (from 0 to 11)	0.02* (0.01) **[0.00; 0.04]**	0.01 (0.01) **[-0.00; 0.03]**
**Physical functioning subscale of the SF-36** (from 0 = worst to 100 = best)	0.00*** (0.00) **[0.00; 0.00]**	0.00 (0.00) **[-0.00; 0.00]**
**Constant**	1.78*** (0.33) **[1.12; 2.43]**	2.86*** (0.28) **[2.32; 3.42]**
**Observations**	4,264	4,255
**Number of individuals**	2,439	2,441
**R²**	0.07	0.06

Unstandardized beta-coefficients are displayed. Robust standard errors in parentheses(), and 95% Confidence intervals in []; *** p < 0.001, ** p < 0.01, * p < 0.05, + p < 0.10; CES-D = The Center for Epidemiological Studies Depression Scale, Ref = Reference group, MET = metabolic equivalent task

## Discussion

### Key results

Based on nationally representative data, the aim of this study was to investigate longitudinal associations between physical activity and perceived exclusion and loneliness in middle-aged adults. Our results revealed that stopping to follow the WHO physical activity recommendations was associated with an increased feeling of being socially excluded among middle-aged. This current longitudinal study adds to current knowledge by addressing middle-aged adults, rather than older adults and adolescence.

### Interpretation

In contrast to some previous cross-sectional studies, we did not find a significant negative association between physical activity and loneliness [10, 35–37]. Statistically significant estimates were observed for stopping to follow the WHO physical activity recommendations and perceived social exclusion. In consequence, taking the heterogenous study results into account it remains uncertain if there is an association between physical activity and loneliness and performing a meta-analysis on the association is indicated. This is the first study examining the association between physical activity and perceived social exclusion. However, to be certain about the association future studies should be conducted. Finally, the effect estimate is small (0.09 on the Bude Latermann scale ranging from 1 to 4). If it is clinically relevant needs as well further investigation, since a clinically meaningful change for this tool has to the best of our knowledge not been identified yet.

Our finding that stopping to follow the WHO recommendations was associated with an increase in perceived social exclusion but not with an increase in loneliness which might be explained by the underling age range of the sample. First, middle-aged adults decreased their physical activity likely due to some specific mechanisms. One mechanism could be: time restrictions due to family responsibilities, which might lead to less physical activity in groups and clubs, where the individuals became part of the society. Thus, stopping to participating in a physical activity organised for the society, may in particular negatively influence the feeling of social exclusion. The feeling of loneliness could in the given example remain unchanged, since taking up responsibilities for the family might support the quantity and quality of the relationship within the family. This explanation holds also true if the middle-aged individual stopped participating in a sports group due to heath issues. The chance that the connections in the social network of the person remain or even increase is possible and likely since most friends will be able to adopt and if needed visit the person at home. This ability of adopting to a new way of meeting with friends is no longer given in older adults, if an elderly loses the ability to visit friends in nursing homes the friends might physically not be able to adopt.

Future studies on middle aged-adults should consider the context and type of the physical activity. If adults stopped participating at sport groups, such as a spinning course (for the society) this could explain our observed difference in the association between physical activity and perceived social exclusion and loneliness.

The importance of the underling activity could also explanation why starting to follow the WHO physical activity recommendations was not associated with our outcome measures. Even if middle-aged adults start to join a sports group, and increase their physical activity, the quality of relationships might be more important than the quantity in this age bracket, and most high-quality relationships need time to be established thus it is unlikely to be associated with loneliness [38]. The same holds true for perceived social exclusion, to feel part of the society or a sport groups is likely to need some time.

### Strengths and Limitations

Some limitations to this study need to be mentioned. We cannot dismiss the possibility that reverse causality exists (e.g., from loneliness to physical activity). A reversed causality could be explained by the Social Control Theory of Hirschi, which states that the social environment is at least partly controlling an individual’s behaviour [39]. Thus, if an individual lacks friends and family, who are often motivators or role models for trying something new – a lonely individual is less likely to engage in any (physical) activity. However, Kwag et al. [40] suggests that physical activity contributes to loneliness, and thus assumes a similar causality direction as we do. Consequently, the causality between physical activity and loneliness as well as perceived social exclusion needs further investigations using longitudinal data.

Measuring physical activity levels is particularly challenging in large national studies, since applying the golden standard accelerometer is not practicable [41–43]. Hence, all our measurement tools were self-reported, which introduces the chance of biases. However, in investigation within individuals, biases are reduced, since it is likely that individuals constantly overestimate physical activities over time [44]. In addition, we used a physical activity measurement tool which was suggested for studying within-individual associations [42, 45].

Another limitation arises from the available and used data. We could only use information from two time points since the information on PA was first added to the DEAS in 2014. This leads, however, to a lack of information on the possible long term fluctuation in the association between our investigated variables. Furthermore, we adjusted our analyses for ten time-varying variables since their omission would have potentially let to spurious effects. Nonetheless, there is still the chance that unobserved time-varying variables influenced our results. Therefore, we suggest performing further studies to verify the observed association between physical activity and perceived social exclusion.

Finally, this is not a prediction model study, but a prognostic factor study. Thus, the study results inform about an association and should not be used to predict changes in perceived social exclusion and loneliness.

One major strength of the present study was the data source, which enables longitudinal investigations based on a representative sample. The fixed effects regressions are another strength of this study. The use of this method reduced the influence of unobserved variables on the estimates since constant variables do not bias the estimates. Finally, the asymmetric fixed effects regressions assist to shed light on the associations between beginning as well as stopping physical activities (according to the WHO guidelines) and the outcome measures. Presenting unadjusted and adjusted model results, is another strength of our article since it supports transparency on the performed analyses.

## Conclusion and future research

Longitudinal associations between physical activity and perceived social exclusion were identified in middle-aged adults. Health professionals targeting perceived social exclusion should thus amongst others consider the contribution of maintaining to follow the WHO physical activity recommendations of at least 150 min of moderate physical activity per week as part of their treatment plan. To support the evidence regarding causality, future research should also investigate how changes in physical activity in middle-aged adults contribute to overall health later in life. Furthermore, the potentially moderating role of the type of activity, the preference, and the satisfaction with performed physical activities in the association between physical activity and loneliness/perceived social exclusion should be investigated in future studies. Furthermore, factors impacting the PA levels in middle aged adults such as self-efficacy, and employment status should be considered. Such information would be valuable to target reduced physical activity levels in this age group.

## Data Availability

The anonymised data used in this study were obtained from the “Deutsches Zentrum für Altersfragen”. Access can be obtained after application, please visit the following website for further information Access to DEAS data: Deutsches Zentrum für Altersfragen (dza.de).
